# Key m^6^A regulators mediated methylation modification pattern and immune infiltration characterization in hepatic ischemia-reperfusion injury

**DOI:** 10.1186/s12920-023-01751-0

**Published:** 2023-12-04

**Authors:** Yixi Zhang, Can Qi, Yiwen Guo, Xuefeng Li, Zebin Zhu

**Affiliations:** 1 Department of General Surgery, Beijing Friendship Hospital, Capital Medical University, NO. 95 Yongan Road, Beijing, 100051 China; 2 Organ Transplant Center, The First Affiliated Hospital of USTC, Division of Life Sciences and Medicine, University of Science and Technology of China, NO.17 Lujiang Road, Hefei, 230001 Anhui China; 3 Organ Transplant Centre, The First Affiliated Hospital, Sun Yat-sen University, NO. 58 Zhongshan Er Road, Guangzhou, 510080 Guangdong China

**Keywords:** Hepatic ischemia-reperfusion injury, m^6^A methylation regulators, YTHDC1, METTL3

## Abstract

**Background:**

N^6^-methyladenosine (m^6^A) mRNA modification plays a critical role in various human biological processes. However, there has been no study reported to elucidate its role in hepatic ischemia-reperfusion injury (IRI). This study was aimed to explore the expression pattern together with the potential functions of m^6^A regulators in hepatic IRI.

**Methods:**

The gene expression data (GSE23649) of m^6^A regulators in human liver tissue samples before cold perfusion and within 2 h after portal vein perfusion from Gene Expression Omnibus database was analyzed. The candidate m^6^A regulators were screened using random forest (RF) model to predict the risk of hepatic IRI. The evaluation of infiltrating abundance of 23 immune cells was performed using single sample gene set enrichment analysis. Besides, quantitative real time polymerase chain reaction (qRT-PCR) assay was carried out to validate the expression of key m^6^A regulators in mouse hepatic IRI model.

**Results:**

The expressions of WTAP, CBLL1, RBM15, and YTHDC1 were found to be increased in liver tissues 2 h after portal vein perfusion; in contrast, the expressions of LRPPRC, FTO, METTL3, and ALKBH5 were decreased. Based on RF model, we identified eight m^6^A methylation regulators for the prediction of the risk of hepatic IRI. Besides, a nomogram was built to predict the probability of hepatic IRI. In addition, the levels of WTAP, ALKBH5, CBLL1, FTO, RBM15B, LRPPRC and YTHDC1 were correlated with the immune infiltration of activated CD4 T cell, activated dendritic cell (DC), immature DC, mast cell, neutrophil, plasmacytoid DC, T helper (Th) cell (type 1, 2, and 17), gamma delta T cell, T follicular helper (Tfh) cell, myeloid-derived suppressor cell (MDSC), macrophage, natural killer cell, and regulatory Th cell. Among mouse hepatic IRI model, the mRNA level of CBLL1 and YTHDC1 was increased with statistical significance; however, the mRNA level of FTO and METTL3 was decreased among post-reperfusion liver samples compared with those in pre-reperfusion samples with statistical significance.

**Conclusions:**

The m^6^A regulators exerted a pivotal impact on hepatic IRI. The m^6^A patterns that found in this study might provide novel targets and strategies for the alleviation/treatment of hepatic IRI in the future.

**Supplementary Information:**

The online version contains supplementary material available at 10.1186/s12920-023-01751-0.

## Background

Hepatic ischemia-reperfusion injury (IRI) is one of the most important causes of liver dysfunction and a severe complication of liver surgery or traditional liver transplantation. Moreover, this damage was linked with the quality of allografts as well as the poor outcomes of patients with liver transplantation. How to mitigate hepatic IRI following liver transplantation has always been the focus of researches. A variety of factors, including mitochondrial damage, oxidative stress, reactive oxygen species (ROS) release, anaerobic metabolism, intracellular Ca^2+^ overload, cytokines and chemokines produced by Kupffer cells and neutrophils (NEUs), as well as NO are involved in the regulation of the process of hepatic IRI [1]. At present, RNA modifications during organ IRI, especially N6-methyladenosine (m^6^A), have been drawing increasing attentions [2].

As the most abundant mRNA modification in eukaryotic cells, m^6^A is deposited by the m^6^A methyltransferase complex consisted by METTL3/14/16, WTAP, KIAA1429 and RBM15/15B, erased by demethylases (FTO and ALKBH5), and recognized by binding proteins (e.g., YTHDF1/2/3, YTHDC1/2 and IGF2BP1/2/3) [3]. As reported, m^6^A RNA modifications exerts a critical effect on organ IRI, for example, age-related differences in m^6^A RNA methylation were found in acute myocardial IRI [4]; in mice, m^6^A modifications were found to be increased in 122 mRNAs together with 17 long noncoding RNAs (lncRNAs), but decreased in 15 mRNAs together with 3 lncRNAs in the ischemic brain at 12 hours reperfusion [5]; Xu et al. demonstrated the regulating role of m^6^A RNA methylation in the development/progression of renal IRI [6]. However, none of the studies have yet explored the effect of m^6^A on hepatic IRI. Consequently, our study was purposed to summarize the implications of m^6^A regulators in the liver for hepatic IRI.

In the present study, using Gene Expression Omnibus (GEO) database, the differentially expressed (DE)- regulators of m^6^A methylation were analyzed in the samples of liver tissues before cold perfusion and within 2 h after portal vein perfusion. The performance of the support vector machine (SVM) as well as random forest (RF) methods in predicting the probability of hepatic IRI was compared. Additionally, we evaluated the effect of DE- m^6^A methylation regulators on immune cell infiltration (ICI). Moreover, to validate the reliability of our bioinformatics analysis, the expressions of key m^6^A methylation regulators were detected by quantitative real time polymerase chain reaction (qRT-PCR) in mouse hepatic IRI model in vivo.

## Methods and materials

### Data acquisition

First, GSE23649 dataset was downloaded from GEO (https://www.ncbi.nlm.nih.gov/geo/), which is an openly accessible database. The raw data of GSE23649 dataset containing 33 liver samples was taken immediately before cold perfusion (baseline); besides, 33 liver samples that taken within 2 h after portal reperfusion and 3 samples form living donors that taken at the start of surgery are included as controls or reference samples. Then, based on previous literatures [7, 8], twenty-six regulators of m^6^A methylation were extracted to identify the patterns of m^6^A regulator-mediated modification, including 9 writers (WTAP, ZC3H13, CBLL1, METTL3, METTL14, METTL16, VIRMA, RBM15 and RBM15B), 15 readers (ELAVL1, FMR1, LRPPRC, YTHDC1, YTHDC2, YTHDF1, YTHDF2, YTHDF3, HNRNPC, HNRNPA2B1, IGFBP1, IGFBP2, IGFBP3, IGF2BP1 and RBMX) as well as 2 erasers (ALKBH5 and FTO).

### RF analysis for the screening of the feature genes

**Fig. 1:**
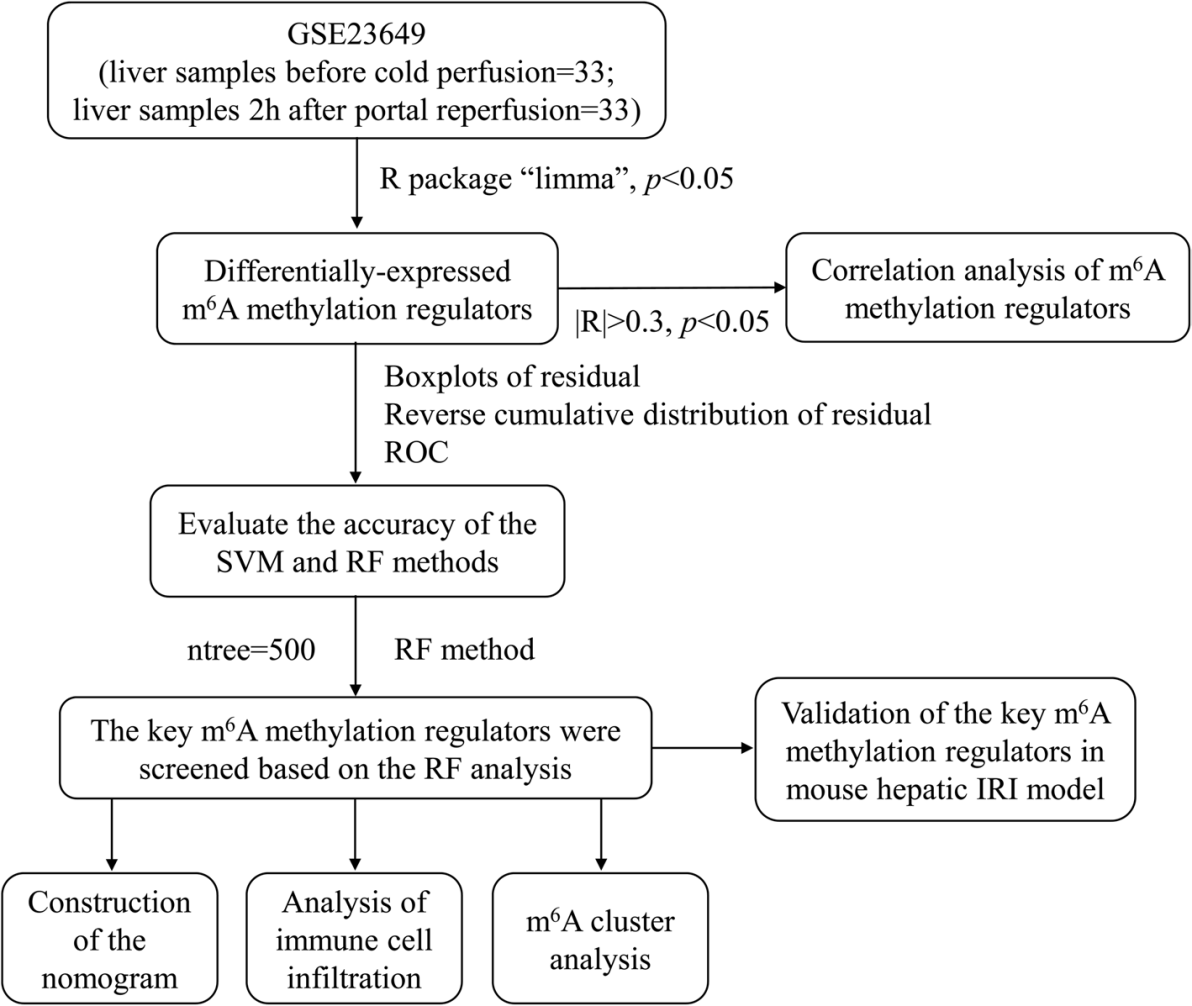
A flow chart for data processing and analysis. m^6^A, N^6^-methyladenosine; ROC, receiver operating characteristic; IRI, ischemia-reperfusion injury

We followed the flow chart shown in Fig. 1 for data processing and analysis. DE- m^6^A methylation regulators between liver samples before cold perfusion (control) and liver samples 2 h after portal were screened via the “limma” R package, with *p* < 0.05 as the criterion for DE- genes. Pearson correlation coefficient analysis was performed for the DE- regulators of m^6^A methylation using |R| > 0.3 and *p* < 0.05 as the criteria.

SVM as well as RF were used for the construction of a training model to predict the occurrence of hepatic IRI. In addition, to assess the accuracy of the constructed model, we used residual boxplot, inverse cumulative distribution of residual along with receiver operating characteristic (ROC) curve. DE- regulators of m^6^A methylation were analyzed to predict the hepatic IRI by RF method, and calculated by R library “randomForest”. In our analysis, the ntree was set to 500. The optimal ntree was chosen on the basis of the minimum cross-validation (CV) error in the 10-fold CV. Besides, the significance of DE- regulators of m^6^A methylation was assessed using the optimal ntree. Then, the m^6^A methylation regulators with importance scores greater than 2 were identified. The “rms” software package was used for nomogram construction. Calibration curves were adopted to assess whether the actual observed value was in agreement with the predicted values. Additionally, the clinical impact curve as well as decision curve analyses were used for the evaluation of clinical benefit of the constructed model.

### Analysis of m^6^A clusters as well as estimation of ICI

Consensus clustering using the ConsensusClusterPlus package in R software was adopted for the identification of m^6^A clusters using the regulators of m^6^A methylation. Besides, in hepatic IRI, the evaluation of abundance of 23 immune cells, the exploration of the correlations, and the construction of bar charts for visualization of features were conducted using single-sample gene set enrichment analysis (ssGSEA).

### Mouse model of hepatic IRI

Mice that underwent hepatic IRI surgery, partial (70%) hepatic ischemia were induced in our study. Briefly, mice were intraperitoneally anesthetized with 1% pentobarbital sodium (Sigma-Aldrich, Saint Louis, USA). Following the midline laparotomy, an atraumatic clip (Fine Science Tools, Foster City, CA, USA) was placed across the portal vein, hepatic artery and bile duct to interrupt blood supply to the left lateral and median lobes (approximately 70%) of the liver and removed after 90 min of partial hepatic ischemia to initiate reperfusion. The mice were anesthetized 6 h following reperfusion, and then samples of ischemic together with non-ischemic lobes (right lateral lobes, served as the controls) were immediately collected for further analyses. Sham-operated mice underwent the same protocol except vascular occlusion, and served as a control group. There were 10 animals in each group.

All the experiments were carried out complying with the Guideline for Laboratory Animals of Capital Medical University. The mice were purchased from Shanghai Model Organisms Center, Inc. (Shanghai, China). All the mice used in this study had a C57BL/6 J genetic background (male, 12–14 weeks old). All the protocols of animal studies were approved by the Ethics Committee of Capital Medical University (Certification number SCXK-JING 2016–0002).

### Quantitative **real time** PCR analysis (qRT-PCR)

The extraction of total RNA was conducted using Trizol (Invitrogen, 15,596,018) complying with the instructions provided by the manufacturer. The preparation of cDNA was carried out via reverse transcription using SuperScript III Reverse Transcriptase (Thermo Fisher Scientific, 18,080,093). SYBR Green PCR Master Mix (Thermo Fisher Scientific, 4,309,155) was used in a 7500 Fast Real-time PCR system (Applied Biosystems, US) for qRT-PCR. Besides, glyceraldehyde-3-phosphate dehydrogenase (GAPDH) was used as an internal control for standardization. The ΔΔCT method was adopted to calculate the normalized value for each target gene. The relative expression of target gene and GAPDH mRNA was compared and expressed as mean ± standard deviation (SD) of fold change relative to the control. The sequences of the primers were presented in Table S1.

### m^6^A methylation quantification assay

Total RNA was extracted from mouse liver tissues using the above method, and m^6^A RNA methylation quantification kit (Abcam, ab185912, UK) was used to detect the total levels of m^6^A RNA methylation in post-reperfusion liver samples and control liver tissues. Experiments were performed according to the manufacturer’s protocol, in brief, 200 ng of sample RNA and 80 μL of binding solution were added to 96-well plates and incubated at 37 °C for 90 min. The samples were then incubated with the capture antibody for another 60 min and finally incubated with the developer solution for 10 min. Absorbance values were measured at a wavelength of 450 nm, and the analyses of relative quantification of m^6^A was performed.

### Immunofluorescence staining

Immunofluorescence staining was used to detect the infiltration of various immune cells in the liver tissues. The expression of F4/80, Ly6G, tryptase, CD3 and CD11b was measured to assess the infiltration status of macrophages, NEUs, mast cells (MCs), T lymphocytes and dendritic cells (DCs), respectively. Briefly, after deparaffinisation and antigen retrieval, paraffin-embedded liver tissue sections were incubated in 3% hydrogen peroxide for 15 minutes to quench endogenous peroxidase activity. The sections were then permeabilized with 0.2% Triton X-100 (Abcam, UK) for 15 min and blocked with 5% BSA (Abcam, UK) for 30 min. Incubation of primary antibodies was performed, antibodies against CD3 (1:300, Abcam, ab135372, UK), CD11b (1:300, Abcam, ab8878, UK), Ly6G (1:100, Abcam, ab303467, UK), tryptase (1:100, Santa Cruz Biotechnology, sc-59,587, Germany) and F4/80 (1:300, Abcam, ab6640, UK), overnight at 4 °C. The tissue sections were then treated with secondary antibodies (1:500, Cell Signaling Technology, USA) for 30 min at room temperature. Finally, a 4, 6-diamidino-2-phenylindole (DAPI) staining solution (Beyotime Biotechnology, China) was used to counterstain the nuclei. The tissue sections were observed and captured with an inverted fluorescence scanning microscope (Nikon Eclipse Ti-SR, Japan).

### Statistical analysis

Statistical analysis was performed by R software (version 4.2.1, http://www.r-project.org). Differences between groups were compared using Student’s *t*-test. Besides, differences in ≥3 groups were compared using one-way analysis of variance (ANOVA). Correlation of gene expression levels were explored by Pearson’s correlation test. A two-tailed *p*-value < 0.05 indicates a statistical significance.

## Results

### Correlation of m^6^A RNA methylation with hepatic IRI

**Fig. 2:**
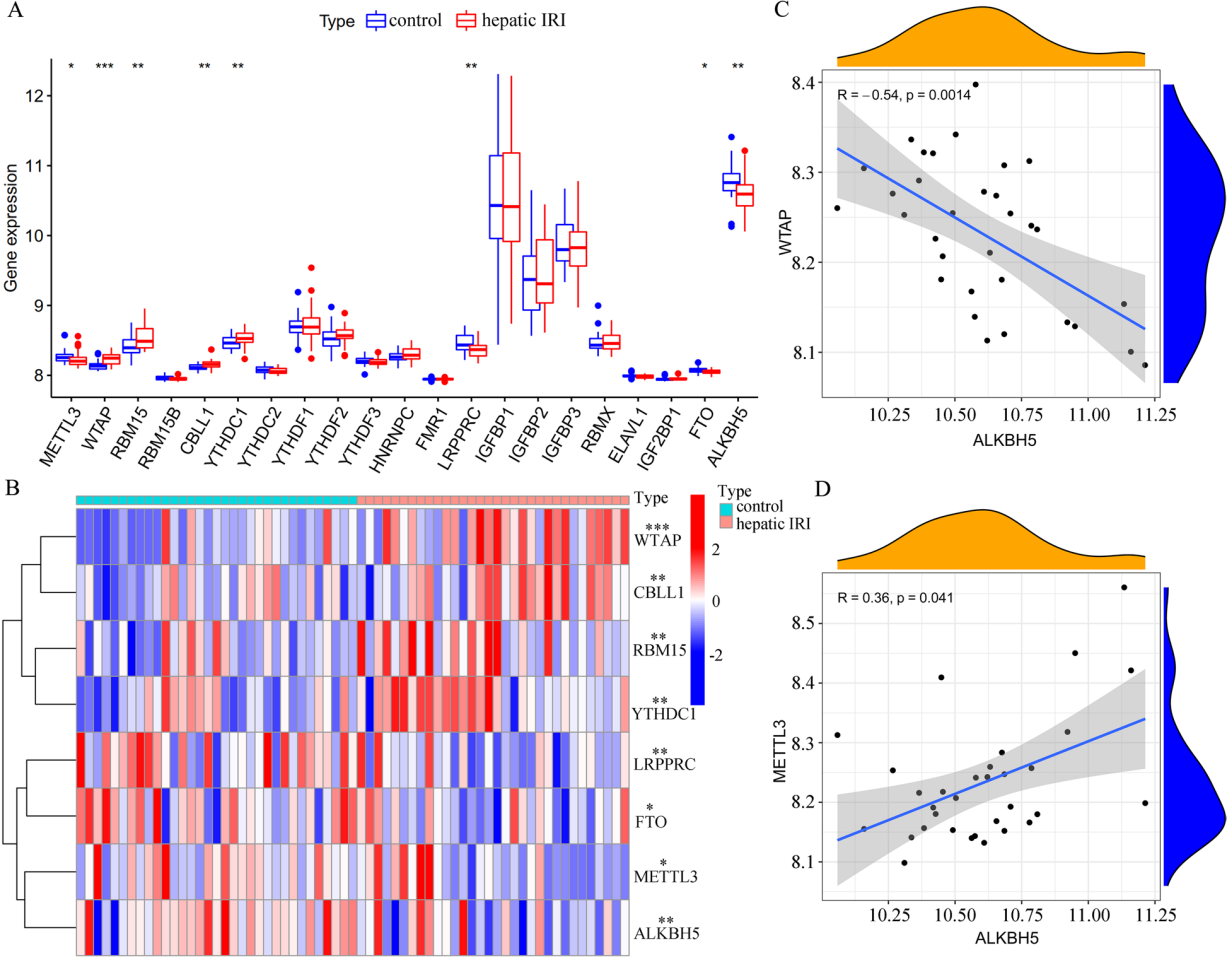
Relationship of m^6^A RNA methylation with hepatic IRI. **A** Box plots showed the differential gene expression between liver tissues before cold perfusion (*n* = 33) and liver tissues 2 h after portal vein perfusion (*n* = 33). **B** Heat map showed the significantly different expressions of the 8 DE- regulators of m^6^A methylation. **C** Correlation analysis between WTAP and ALKBH5 expressions. **D** Correlation analysis between METTL3 and ALKBH5 expressions. *p < 0.05, **p < 0.01, ***p < 0.001

To find out the hepatic IRI-associated changes in m^6^A methylation, we performed differential expression analysis for m^6^A methylation regulators. First, based on GSE23649 dataset, DE- m^6^A methylation regulators were analyzed in liver tissue samples before cold perfusion (*n* = 33) and 2 h after portal vein perfusion (*n* = 33). WTAP, CBLL1, RBM15, as well as YTHDC1 were found to be highly expressed in liver tissues 2 h after portal vein perfusion. In contrast, LRPPRC, FTO, METTL3, and ALKBH5 showed the opposite results (Fig. 2A). In addition, a heat map was constructed to display these DE- genes (Fig. 2B). Correlation analysis based on the GSE23649 dataset also showed that the expression level of WTAP was negatively related to that of ALKBH5 (R = − 0.54, *p* = 0.0014, Fig. 2C), and the expression level of METTL3 was positively related to that of ALKBH5 (R = 0.36, *p* = 0.041, Fig. 2D).

### Construction of hepatic IRI predictive models using SVM and RF methods

**Fig. 3:**
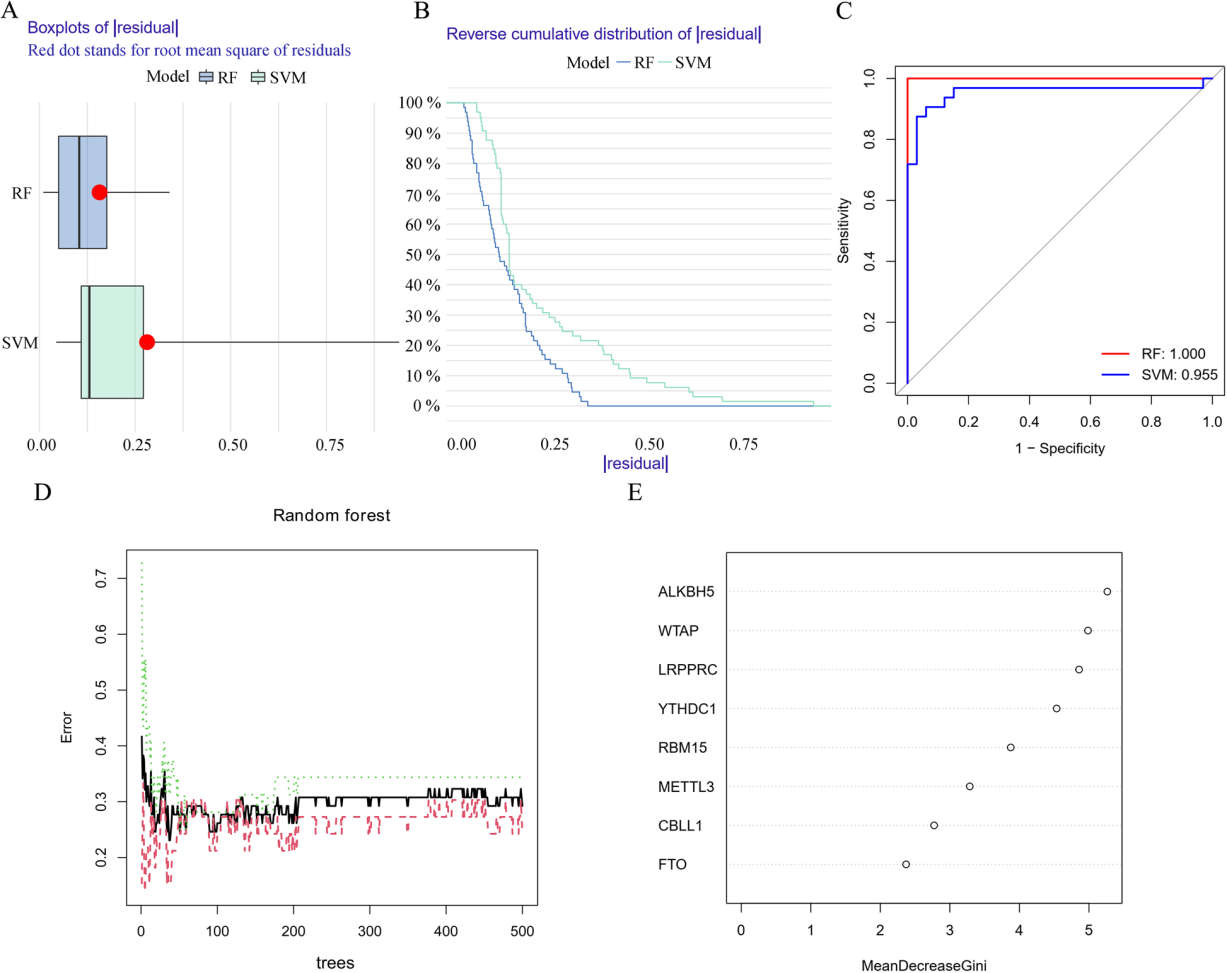
Construction of hepatic IRI predictive models using SVM and RF methods. Boxplots of the residual distribution (**A**) together with the reverse cumulative distribution of residual (**B**) as the observed sensitivity between RF and SVM models. **C** ROC curves for RF and SVM models. **D** RF: Prediction error curves using 10-fold cross-validation. **E** Importance of the eight m^6^A regulators on the basis of RF model. RF, random forest; SVM, support vector machine

First, the predictive performance of SVM was compared with RF model. The residual boxplot (Fig. 3A), inverse cumulative distribution of residuals (Fig. 3B), and ROC curve (Fig. 3C) all indicated that RF method had high prediction accuracy. The minimum residuals of all samples in the model indicate that the model is optimal. The selection of optimal ntree was carried out using the smallest CV error in the 10-fold CV (Fig. 3D). Genes were ranked by the importance, and then eight m^6^A methylation regulators were identified (Fig. 3E).

**Fig. 4:**
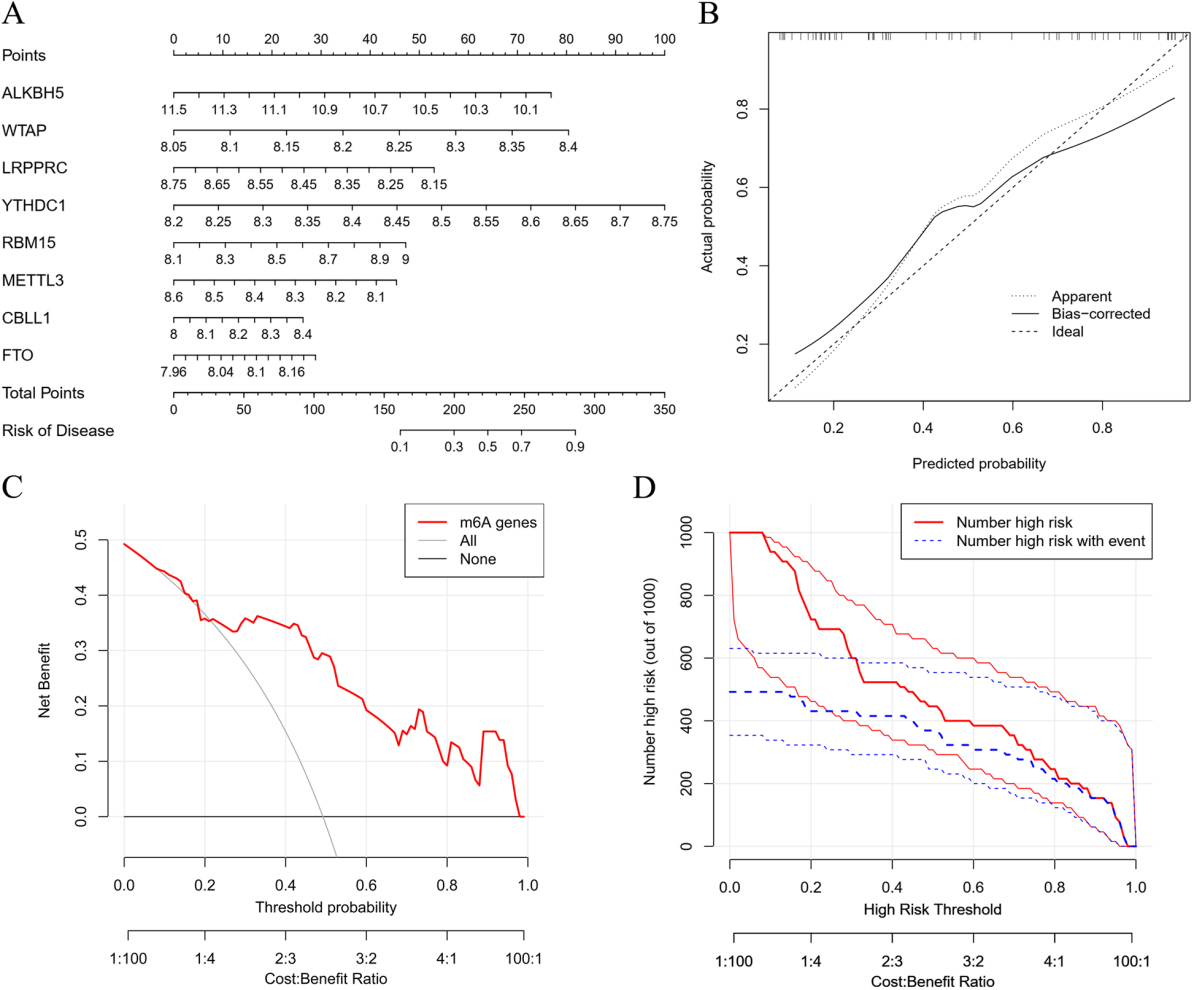
Construction of the nomogram predicting the probability of hepatic ischemia-reperfusion injury. **A** A nomogram for the prediction of the probability of hepatic IRI based on the eight m^6^A regulators. **B** The calibration curves proved that the nomogram might be ideal for the prediction of hepatic IRI. **C, D** DCA plot and clinical impact plot were applied to determine the clinical utility of the constructed nomogram. DCA, decision curve analysis

Next, based on the eight m^6^A methylation regulatory factors, we built a nomogram evaluation model for the prediction of the probability of liver IRI (Fig. 4A). Based on calibration curve, the nomogram model was found to be ideal for the prediction of hepatic IRI (Fig. 4B). Decision curve analysis as well as clinical impact maps were applied for the determination of the clinical utility of the nomogram (Fig. 4C and D). These results suggested a good predictive ability of the nomogram model for hepatic IRI.

### Identification of m^6^A clusters as well as analysis of ICI

**Fig. 5:**
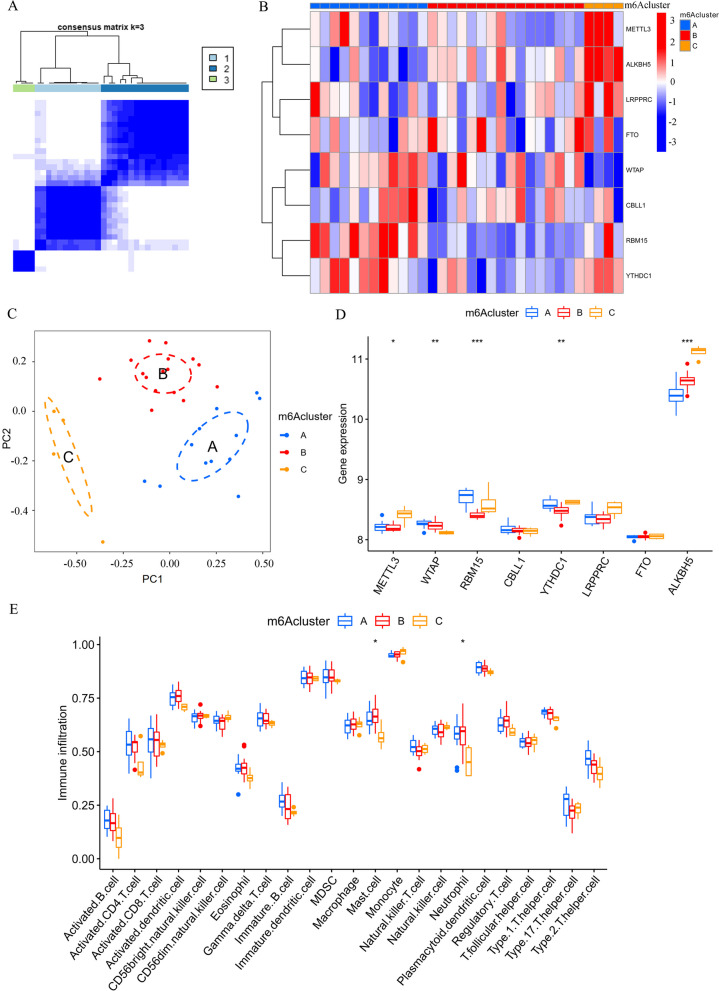
Identification of m^6^A clusters as well as analysis of immune cell infiltration. **A** Matrix of consensus clustering of liver tissue samples 2 h after portal vein perfusion for k = 3. **B** The liver tissues 2 h after portal vein perfusion were divided into 3 clusters using consensus clustering (clusters A/B/C). A heat map was applied to show differential gene expressions of the three m^6^A gene clusters. **C** The 3 distinct m^6^A clusters were verified by PCA. **D** The expressions of METTL3, WTAP, RBM15, YTHDC1 and ALKBH5 were identified among the 3 m^6^A clusters. **E** Box plots showed the immune cells infiltrating in the 3 m^6^A clusters. *p < 0.05, **p < 0.01, ***p < 0.001; PCA, principal component analysis

In total, 3 m^6^A clusters (A/B/C) were identified based on the 8 m^6^A methylation regulators using consensus clustering method (Fig. 5A). Figure 5B shows the heat map of differential gene expression among the 3 m^6^A clusters. Principal component analysis was adopted to validate the 3 different m^6^A clusters divided by consensus clusters of 5 regulators of m^6^A RNA methylation (Fig. 5C). In addition, differences with statistical significance were found in the expressions of METTL3, WTAP, RBM15, YTHDC1 and ALKBH5 among the 3 m^6^A clusters (Fig. 5D). Based on the substantial ICI in hepatic IRI [9], the immune cells infiltrating between m^6^A clusters were further analyzed. There were differences of the infiltration of MCs along with NEUs found among the m^6^A clusters with statistical significance (Fig. 5E).

### Relationship of key m^6^A methylation regulators with ICI

**Fig. 6:**
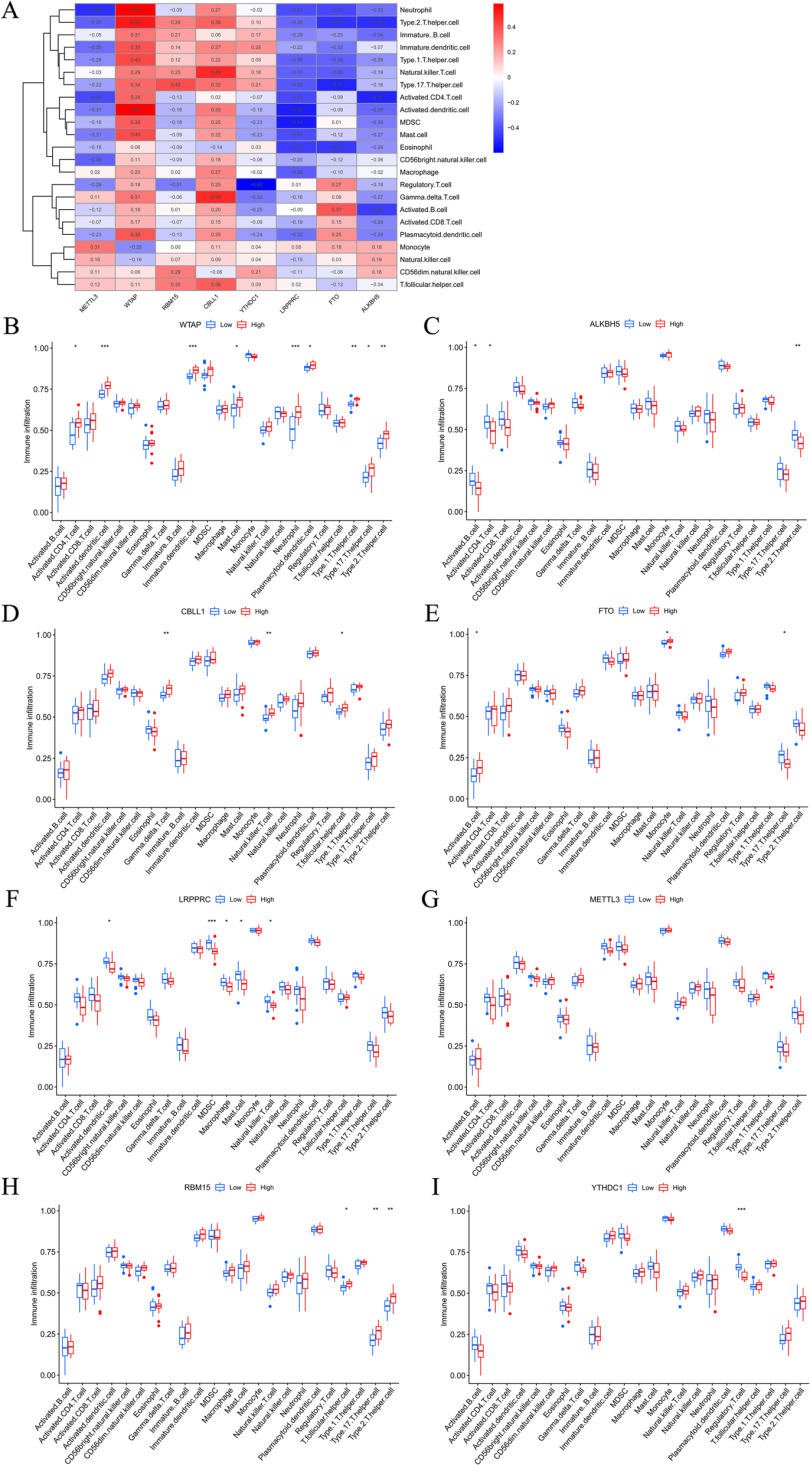
Relationship of the eight key m^6^A methylation regulators with immune cell infiltration. **A** Heat map for the correlation of the expressions of the eight regulators of m^6^A methylation with the infiltration of immune cells using single-sample gene set enrichment analysis. **B** Infiltration profile of 23 immune cells in high−/low- (H−/L-) WTAP expression groups. **C** Infiltration profile of 23 immune cells in H−/L- ALKBH5 expression groups. **D** Infiltration profile of 23 immune cells in H−/L- CBLL1 expression groups. **E** Infiltration profile of 23 immune cells in H−/L- FTO expression groups. **F** Infiltration profile of 23 immune cells in H−/L- LRPPRC expression groups. **G** Infiltration profile of 23 immune cells in H−/L- METTL3 expression groups. **H** Infiltration profile of 23 immune cells in H−/L- RBM15 expression groups. **I** Infiltration profile of 23 immune cells in H−/L- YTHDC1 expression groups. *p < 0.05, **p < 0.01, ***p < 0.001

Next, whether the expressions of the 8 regulators of m^6^A methylation were related with ICI were investigated using ssGSEA (Fig. 6A). In the high WTAP expression group, the immune infiltration of activated CD4 T cell, activated DC, immature DC, MC, NEU, plasmacytoid DCs, T helper (Th) cells (type 1, 2, and 17) was significantly increased (Fig. 6B). The immune infiltration of activated B cells, activated CD4 T cells as well as type 2 Th cells was significantly reduced in high ALKBH5 expression group (Fig. 6C). In the high CBLL1 expression group, the immune infiltration of gamma delta T (GDT) cells, natural killer (NK) T cells and T follicular helper (Tfh) cells was significantly increased (Fig. 6D). In the high FTO expression group, the immune infiltration of activated B cells and monocyte was significantly increased, while the immune infiltration of type 17 Th cell was decreased with statistical significance (Fig. 6E). In the high LRPPRC expression group, the immune infiltration of activated DCs, myeloid-derived suppressor cells (MDSCs), macrophages, MCs and NK cells were significantly reduced (Fig. 6F). The immune infiltration of the 23 immune cells was not significantly different between the high and low METTL3 expression groups (Fig. 6G). In the high RBM15 expression group, the immune infiltration of Tfh cell and Th cell (type 2 and 17) was significantly increased (Fig. 6H). The immune infiltration of regulatory Th cell was significantly reduced in the high- YTHDC1 expression group (Fig. 6I).

### Validation of the 8 key m^6^A regulators in mouse hepatic IRI model

**Fig. 7:**
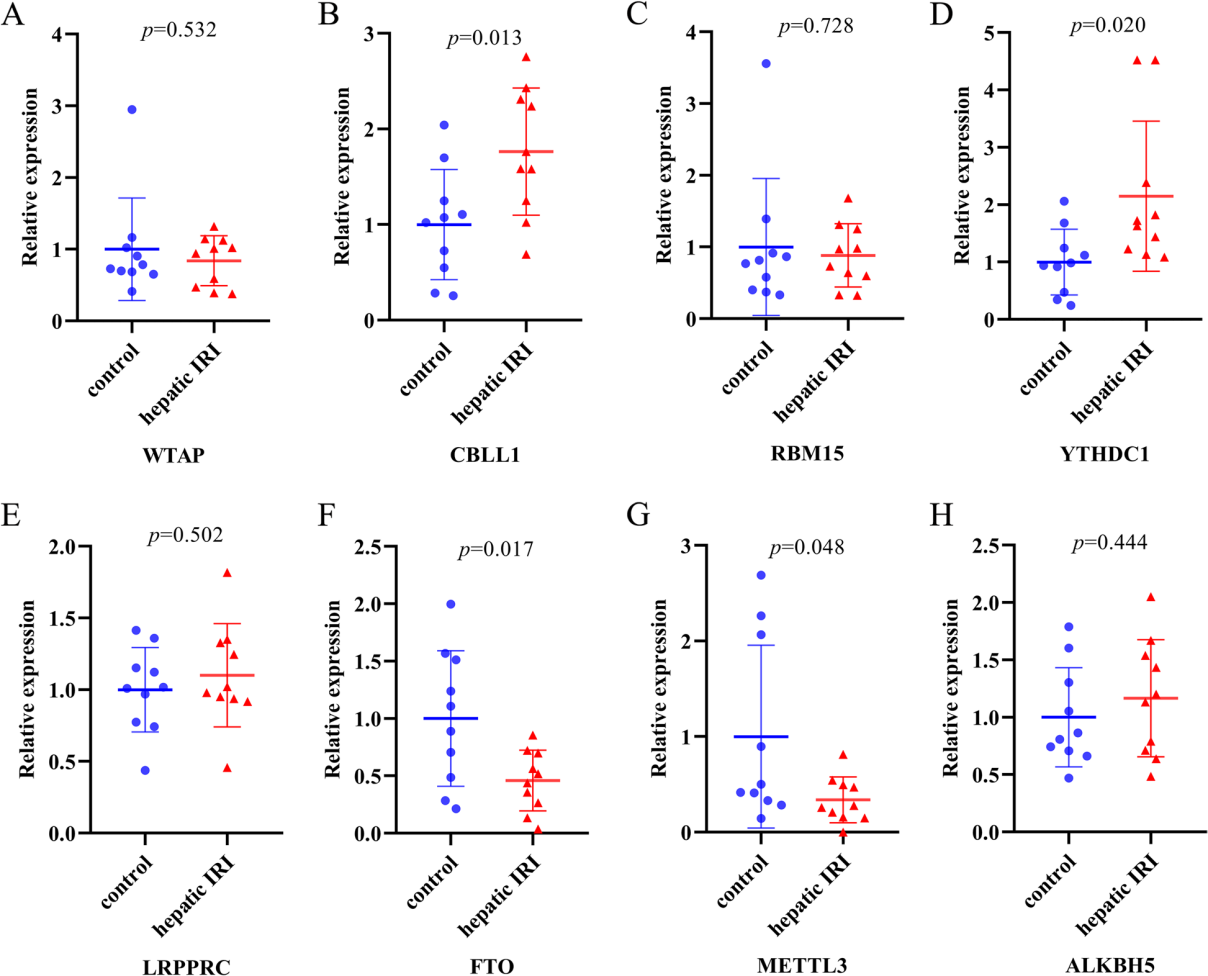
qRT-PCR validation of the 8 differentially-expressed m^6^A methylation regulators among mouse hepatic IRI model. **A-H** The relative mRNA expressions of WTAP, CBLL1, RBM15, YTHDC1, LRPPRC, FTO, METTL3, and ALKBH5 in post-reperfusion liver samples and control samples (*n* = 10 per group), unpaired *t* test was adopted for statistical analysis

To determine the 8 key m^6^A regulators correlated with liver IRI, qRT-PCR was adopted to detect the expressions of the 8 key m^6^A regulators in mouse hepatic IRI model. The mRNA expressions of CBLL1 and YTHDC1 were found to be significantly increased in post-reperfusion liver samples compared to the controls, while the expressions of FTO and METTL3 were significantly decreased in post-reperfusion liver samples compared to the controls (Fig. 7). These experimental results were consistent with the above findings in our bioinformatics analysis. However, differences in the expressions of WTAP, RBM15, LRPPRC, and ALKBH5 were not significant between post-reperfusion liver samples and controls.

### Total RNA m^6^A modification level and the immune cells infiltration in mouse hepatic IRI model

**Fig. 8:**
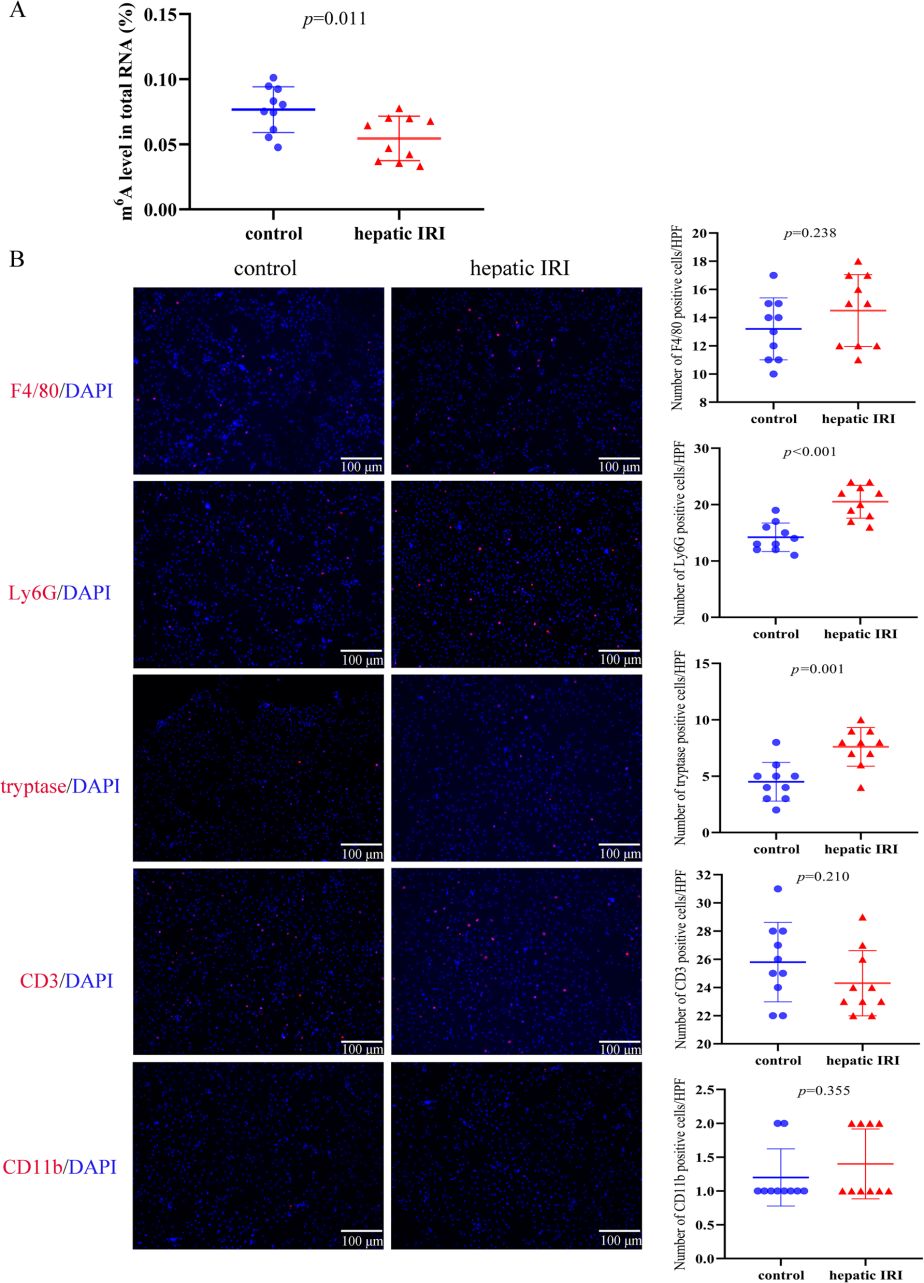
Total RNA m^6^A modification level and the immune cells infiltration in hepatic tissues among mouse hepatic IRI model. **A** The total RNA m^6^A modification level in hepatic tissues were tested by m^6^A methylation quantification assay. **B** Representative immunofluorescence staining of F4/80 (marker of macrophages), Ly6G (marker of neutrophils), tryptase (marker of mast cells), CD3 (marker of T lymphocytes) and CD11b (marker of dendritic cells) in hepatic tissues between the post-reperfusion group and control group. Quantification of F4/80, Ly6G, tryptase, CD3 and CD11b per HPF. Scale bar: 100 μm. *n* = 10 per group, unpaired *t* test was adopted for statistical analysis. DAPI, 4, 6-diamidino-2-phenylindole; HPF, high power field

The m^6^A methylation quantification assay was conducted to assess the RNA m^6^A modification change in hepatic tissues after IRI. The total RNA m^6^A modification level was found to be significantly decreased in post-reperfusion liver samples compared to the controls (Fig. 8A). Additionally, the infiltrating immune cells (macrophages, NEUs**,** MCs, T lymphocytes and DCs) in post-reperfusion liver samples and control samples were detected by immunofluorescence assays. We found that the expression level of Ly6G (a marker of NEUs**)** and tryptase (a marker of MCs) was significantly higher in post-reperfusion liver samples than that in control samples (Fig. 8B)**.** While, there was no significant difference in F4/80 (a marker of macrophages), CD3 (a marker of T lymphocytes) or CD11b (a marker of DCs) expression between the post-reperfusion liver samples and the control samples (Fig. 8B)**.** These results of quantitative analysis of total RNA m^6^A modification and immunofluorescence staining verified the accuracy of the results of our bioinformatics analysis to some extent.

## Discussion

In this study, we found WTAP, CBLL1, RBM15, and YTHDC1 expressions to be increased in liver tissues 2 h after portal vein perfusion compared to that in liver tissues before cold perfusion; in contrast, the expressions of LRPPRC, FTO, METTL3, and ALKBH5 were significantly decreased. We also identified the eight m^6^A methylation regulators for the prediction of risk of hepatic IRI. Besides, a nomogram built on these predictive m^6^A methylation regulators showed good calibration and clinical utility abilities. In addition, based on the 8 m^6^A methylation regulators, three m^6^A clusters were identified. In the distinct m^6^A clusters, significant differences were found in the infiltration of MCs as well as NEUs. Moreover, WTAP, ALKBH5, CBLL1, FTO, RBM15B, LRPPRC and YTHDC1 levels were correlated with the immune infiltration of activated CD4 T cell, activated DC, immature DC, MC, NEU, plasmacytoid DC, Th cell (type 1, 2, and 17), GDT cell, NK T cell, Tfh cell, MDSC, macrophage, NK cell and regulatory Th cell. Importantly, to further validation the data in mouse hepatic IRI model, we found that the expression of either CBLL1 or YTHDC1 was increased; in contrast, the expression of either FTO or METTL3 was decreased in hepatic IRI. Our results proved the importance of m^6^A methylation in the process of hepatic IRI, suggesting it to be a new direction for researches on hepatic IRI.

Growing evidence suggest that m^6^A methylation exerts a critical effect on organ IRI [6, 10, 11], despite of few studies of WTAP, CBLL1, RBM15, and YTHDC1 on IRI. Zhou et al. identified the role of LRPPRC as one of important target genes in *Coptidis rhizoma* alkaloids-mediated protection against cerebral IRI [12]. In cerebral IRI, FTO, ALKBH5 and METTL3 accounted for the abnormal elevated m^6^A modification; besides, FTO overexpression mitigated but ALKBH5 knockdown exacerbated oxygen deprivation/reoxygenation-treated neuronal damage, but [13]. Du et al. demonstrated that during hepatic IRI, FTO was involved in mitochondrial function, and FTO expression was decreased [10]. A recent study also discovered that the expression of FTO was downregulated in myocardial IRI mice as well as hypoxia/reoxygenation (H/R)-induced cardiomyocytes [11]. Meng and his colleagues found that METTL3 protein levels were significantly increased in renal IRI and H/R cell models; in contrast, the inhibition of METTL3 suppressed cell apoptosis in the H/R in vitro model [14]. Conditional knockout of METTL3 from mouse kidneys alleviated ischemia/reperfusion-induced renal dysfunction, injury, or inflammation [15]. Additionally, in myocardial IRI, m^6^A RNA methylation aggravated the injury through ALKBH5-related metabolic reprogramming [16]. Consistent with the results of previous studies, the expressions of LRPPRC, FTO, METTL3, and ALKBH5 were also found to be decreased during hepatic IRI in our study.

The main features of IRI include NEU infiltration along with burst of ROS. Excessive ROS causes oxidative stress in cells and tissues, resulting in the death of cells and eventually the dysfunction of organs. In recent years, some studies have reported that m^6^A is closely related to oxidative stress [17, 18]. In the tumor microenvironment of papillary thyroid carcinoma, METTL3 could regulated the NEU infiltration through its downstream m^6^A target genes [19]. Among clear cell renal cell carcinoma, YTHDF2 expression was demonstrated to be related with the immune infiltration of NEUs [20]. Gong et al. found that the expression levels of m6A methylation transferases had positive correlations with infiltrating levels of NEUs in breast cancer [21]. All these studies suggested that m^6^A methylation played important roles in regulating ICI in tumors. However, in hepatic IRI, how the regulators of m^6^A methylation influence immune infiltration have remained unclear so far. In our study, WTAP was found to be correlated with the immune infiltration of NEUs in the process of hepatic IRI.

During IRI, inflammatory responses might occur in the liver, beginning during hepatic ischemia and aggravating primarily during reperfusion, characterized by the recruitment of numerous NEUs to the liver. Productions of cytokines, chemokines, as well as danger signals cause the activation of resident hepatocytes, leukocytes, together with Kupffer cells. It is now generally accepted that NEUs act as the most important magnifier of liver injury during IRI [9]. In the present study, we also found that there were significant differences in the infiltration of NEUs among the different m^6^A clusters. Interestingly, the immune infiltration of NEU was increased in the high WTAP expression group. Based on these results, it could be inferred that removing excessive NEUs or inhibiting their function by regulating m^6^A RNA methylation during hepatic IRI may could reduce the injury and inflammation in livers.

For T cells, previous studies [22–26] have demonstrated a relevant role of m^6^A in T effector cells, Tfh cells as well as T regulatory cells through the modulation of m^6^A pathway genes in mice. Disruption of m^6^A not only leads to dysregulation of gene expression patterns but also contributes to the development of colitis/systemic autoinflammatory diseases. Recently, the WTAP protein has been proved to be required for the function of the m^6^A methyltransferase complex, and WTAP played a key role in T cell receptor signaling in mouse T cells [27]. Besides, the functions of WTAP together with m^6^A methyltransferase were necessary for thymocyte differentiation, control of peripheral T cell activation-induced death, and prevention of colitis via the activation of intestinal RORγt^+^ regulatory T cell function. In this study, we found that WTAP was linked to immune infiltration of Th cell (type 1, 2, and 17); ALKBH5 was linked to immune infiltration of activated CD4 T cell and Th cell (type 2); CBLL1 was linked to immune infiltration of GDT cell, NK T cell as well as Tfh cell; FTO was linked to immune infiltration of Th cell (type 17); RBM15 was linked to immune infiltration of Tfh cell and Th cell (type 2, and 17); and YTHDC1 was linked to the immune infiltration of regulatory Th cell.

The pivotal finding of our present study is that the key m^6^A regulators mediated the pattern of methylation modification and the characterization of immune infiltration in hepatic IRI. Nevertheless, there are also several limitations here. Firstly, the relatively small sample size was adopted in the original dataset of this study (liver samples before ischemia-reperfusion (*n* = 33) were compared with those after ischemia-reperfusion (*n* = 33)). Randomized controlled studies with a large sample size would be necessary to verify our results in future. Meanwhile, it was a cross-sectional study unable to infer causality. Therefore, our study only showed that the profile of m^6^A methylation could be used as an epigenetic-related marker of the process of hepatic IRI and a possible target for intervention/treatment in future. In addition, although we used in vivo experiments to verify the expression of m^6^A regulators, no profound mechanism was investigated. Thus, more in-depth mechanistic studies on the regulation of m^6^A methylation should be further conducted during hepatic IRI.

## Conclusions

In the study, we identified 8 regulators of m^6^A methylation (WTAP, CBLL1, RBM15, and YTHDC1, LRPPRC, FTO, METTL3 and ALKBH5) were associated with hepatic IRI. In addition, based on the validation using qRT-PCR in mouse hepatic IRI model in vivo, it could be concluded that CBLL1, YTHDC1, FTO and METTL3 played critical roles in hepatic IRI. These candidate m^6^A methylation regulators have a potential to be therapeutic targets for hepatic IRI and further studies are necessary to explore the potential mechanisms of these key biomarkers within the process of hepatic IRI in future.

### Supplementary Information

**Additional file 1.**

## Data Availability

The raw data of GSE23649 dataset was downloaded from GEO (https://www.ncbi.nlm.nih.gov/geo/). The original contributions presented in the study are included in the article. Further inquiries can be directed to the corresponding author.
