# Supplementary material for: Key m6A regulators mediated methylation modification pattern and immune infiltration characterization in hepatic ischemia-reperfusion injury
Source: BMC Med Genomics. 2023 Dec 4;16:314. doi: 10.1186/s12920-023-01751-0 (PMC10694893; doi:10.1186/s12920-023-01751-0)
Supplement: Supplementary file 1 — Additional file 1. [file 12920_2023_1751_MOESM1_ESM.docx]

Table S1. The sequences of all primers used in qRT-PCR

| Gene name | Primer sequence (5'-3') |
| --- | --- |
| WTAP | Forward: 5'-GAACCTCTTCCTAAAAAGGTCCG-3' |
|  | Reverse: 5'-TTAACTCATCCCGTGCCATAAC-3' |
| CBLL1 | Forward: 5'-TCCTTGGGTGGTCTTGATGTT-3' |
|  | Reverse: 5'-CGGCTTAACTTTGCTGGCT-3' |
| RBM15 | Forward: 5'-TTCACGAGTTCAAACGCTTCG-3' |
|  | Reverse: 5'-ACAAAGGCTACCCGCTCATC-3' |
| YTHDC1 | Forward: 5'-GTCCACATTGCCTGTAAATGAGA-3' |
|  | Reverse: 5'-GGAAGCACCCAGTGTATAGGA-3' |
| LRPPRC | Forward: 5'-TTATTGGCATTACCCCTGTCCA-3' |
|  | Reverse: 5'-AAGCTGTCCTATTGGCTTGAAG-3' |
| FTO | Forward: 5'-TTCATGCTGGATGACCTCAATG-3' |
|  | Reverse: 5'-GCCAACTGACAGCGTTCTAAG-3' |
| METTL3 | Forward: 5'-CTGGGCACTTGGATTTAAGGAA-3' |
|  | Reverse: 5'-TGAGAGGTGGTGTAGCAACTT-3' |
| ALKBH5 | Forward: 5'-GCATACGGCCTCAGGACATTA-3' |
|  | Reverse: 5'-TTCCAATCGCGGTGCATCTAA-3' |
| GAPDH | Forward: 5'-TGGCCTTCCGTGTTCCTAC-3' |
|  | Reverse: 5'-GAGTTGCTGTTGAAGTCGCA-3' |

qRT-PCR, quantitative real-time quantitative PCR
